# Safety and efficacy of Empagliflozin in Pakistani Muslim patients with type 2 diabetes (SAFE-PAK); a randomized clinical trial

**DOI:** 10.1186/s12902-022-01213-1

**Published:** 2022-11-28

**Authors:** Azizul Hasan Aamir, Umar Yousaf Raja, Faisal Masood Qureshi, Ali Asghar, Saeed Ahmed Mahar, Ibrar Ahmed, Tahir Ghaffar, Jamal Zafar, Mohammad Imtiaz Hasan, Amna Riaz, Syed Abbas Raza, Irshad Ahmed Khosa, Jahanzeb Khan, Jaffer Bin Baqar

**Affiliations:** 1 Department of Diabetes, Endocrinology and Metabolic Diseases, Khyber Girls Medical College, Hayatabad Medical Complex, Peshawar, Pakistan; 2 Post Graduate Medical Institute, Peshawar, Pakistan; 3 Shifa International Hospital, Islamabad, Pakistan; 4 Al-Khaliq Hospital, Multan, Pakistan; 5 Fatimiyah Hospital, Karachi, Pakistan; 6 National Institute of Cardiovascular Diseases, Karachi, Pakistan; 7 Lady Reading Hospital, Peshawar, Pakistan; 8 Hanif Medical Center, Rawalpindi, Pakistan; 9 Diabetes Institute of Pakistan, Lahore, Pakistan; 10 Jinnah Hospital, Lahore, Pakistan; 11 National Defense Center, Lahore, Pakistan; 12 Balochistan Medical Center, Quetta, Pakistan; 13 Dow University of Health Sciences, Karachi, Pakistan; 14 University of Karachi, Karachi, Pakistan

**Keywords:** SLT2 inhibitors, HbA1c, Safety, Efficacy, Pakistani, Muslims

## Abstract

**Background:**

Sodium-Glucose-Co-Transporter 2 (SGLT2) inhibitor (Empagliflozin) is an effective drug in controlling blood glucose through predominantly glycosuria. Glycosuria increases the risk of genitourinary infections in diabetes. This study was aimed to establish the safety and efficacy of Empagliflozin (Group-A) versus standard care (Group-B) in Pakistani Muslim individuals with type 2 diabetes.

**Methods:**

A multicenter, randomized clinical trial was conducted in five cities across Pakistan from July 2019 to August 2020. Patients of both genders aged 18–75 years, body mass index (BMI) ≤ 45 kg/m^2^, glycosylated hemoglobin (HbA1c) 7–10% (53 mmol/mol to 86 mmol/mol) and treatment-naive to Empagliflozin were included. Treatment was given for 24 weeks, and allocation was done through randomization.

**Results:**

Out of 745 screened patients, 333 met the eligibility criteria, and a total of 244 (73.3%) patients were enrolled. More hypoglycemic events were reported in the standard care group, whereas positive urine culture, fungal infection, dehydration, and hypotension occurrence were comparable between the two groups. The 6 months mean HbA1c reduction was significant in both groups; (Group-A: 0.91 ± 0.15; *p* < 0.001 vs. Group-B2: 0.79 ± 0.14; *p* < 0.001). Efficacy comparison at 6 months revealed a significant reduction in weight and systolic blood pressure (SBP) in Group A only (Group-A: 1.4 ± 0.4 kg; *p* < 0.002 vs. Group-B: 0.01 ± 0.5 kg; *p* < 1.00), (Group-A: 5.1 ± 1.7 mmHg; *p* < 0.012 vs. Group-B: 2.3 ± 1.7 mmHg; *p* < 0.526).

**Conclusions:**

Empagliflozin was a safe drug compared to standard care in Pakistani Muslim patients with diabetes. It was as effective as standard care in the clinical setting but achieved glycemic control by reducing weight and SBP in type 2 diabetes patients.

**Trial registration:**

This study was registered in the NIH US National Library of Medicine clinical trials registry at Clinicaltrials.gov with the registration number: NCT04665284 on 11/12/2020.

## Background

Diabetes is one of the most common non-communicable diseases affecting 463 million adults worldwide. This figure is expected to rise by 2030 to 578 million and 700 million by 2045 [1]. Type 2 Diabetes mellitus (T2DM) is the most common form of diabetes and constitutes almost 90% of the diabetic population. As of 2018, more than 500 million individuals reside with T2DM globally [2]. In Pakistan, the situation is similarly alarming as, according to a recent survey, 16.98% of the adult Pakistani population has type 2 diabetes [3].

Sodium-Glucose-Co-Transporter 2 (SGLT2) inhibitor, Empagliflozin, with its novel mechanism of action for treating patients with T2DM, has its own set of side effects. Increased urinary glucose losses lead to a higher proportion of urinary tract infections and genital tract mycotic infections, and this has been evident from various studies [4]. Also, there is a high prevalence of urinary tract infections in diabetes patients, which may be asymptomatic [5]. In Southeast Asia, the recently published consensus statement by the South Asian Federation of Endocrine Societies has incorporated sodium-glucose co-transporter 2 inhibitors as monotherapy in type 2 diabetes patients who are intolerant or have any contraindication to metformin therapy. Additionally, drugs belonging to this class are also recommended as combination therapy with other oral hypoglycemic agents and insulin [6].

Empagliflozin, however, has not been studied in the Pakistani population yet. The main aim of this study was to establish the efficacy and safety of Empagliflozin in the optimum control of blood glucose in T2DM. This is the first study of its kind being performed in the Pakistani population. Roughly 24% of the world population and 96% of the Pakistani population is Muslim. We postulate that as Muslims make ablution five times a day, there is a probability of lesser genital infections due to wet hygiene practices compared to the data we already have from the western world. Furthermore, due to intense hot weather in this part of the world, the safety in terms of dehydration was also evaluated.

## Methods

We conducted a multicenter open-label randomized clinical trial to evaluate the safety and Efficacy of Empagliflozin (10/25 mg once daily alone or as an add on therapy) along with standard care as intervention (Group A) versus standard care group without Empagliflozin as control (Group B) in the Pakistani Muslim population with T2DM. Further titration and addition of medications in both groups were at the clinician’s discretion. All consenting Pakistani Muslim male and female, type 2 diabetic patients aged between 18 to 75 years, BMI ≤ 45 kg/m^2^ and HbA1c 7 to ≤10% were enrolled from July 2019 to August 2020, from 12 clinical sites spread across 5 cities of Pakistan, including Karachi (*n* = 2), Lahore (*n* = 3), Islamabad (*n* = 2), Peshawar (*n* = 3), Multan (*n* = 1) and Quetta (*n* = 1) with the primary coordinating site at Peshawar. Most patients appeared for follow-up visits (1st follow-up visit-August 2019; 2nd follow-up visit-October 2019; 3rd follow-up visit-January 2020). After obtaining informed consent from all participants’, data was collected.

The purposive sampling technique followed a meticulous patient selection process; all potential participants underwent screening. Those who were eligible for this study were asked to provide informed consent. Those who agreed to participate in the study were then provided a computer-generated random allocation number. MS Excel was used to assign patients to either treatment groups, i.e. (Group A or Group B).

Primary outcome measures for safety included hypoglycemia (self-reported), hypotension, nocturnal hypoglycemia, as per ADA guidelines [7], dehydration, urinary tract infection, diabetic ketoacidosis, fungal infection, and any other adverse events. Secondary outcome measures for efficacy included changes in HbA1c and fasting blood glucose (FBG) measurements. Other measurements included change in weight (kg), BMI (kg/m^2^) as per WHO classification criteria normal weight (BMI 18.5 to < 25.0), overweight (BMI 25.0 to < 30), obesity (BMI ≥ 30.0) [8], waist circumference (cm), blood pressure (mmHg), changes in lipid levels, Quality of life (QoL) and any other significant finding reported by the patient.

**Fig. 1:**
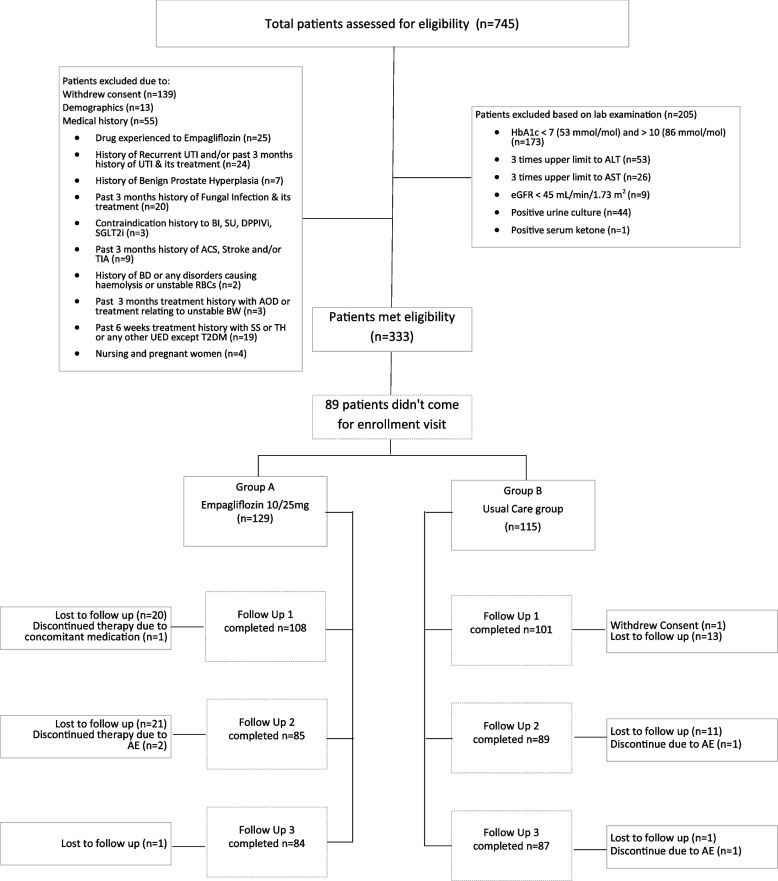
Patient disposition chart of the trial

The sample size (*n* = 328) was calculated using Open Epi sample size estimation for Clinical trials in health studies with 80% power of the test and 95% confidence interval, and proportion of adverse events (45%) [9]. FDA stopping guidelines were utilized based on three ethical scenarios including safety, benefits and futility. A total of 745 patients were assessed for the study eligibility criteria, of which 207 patients were excluded for withdrawing consent (*n* = 139), non-muslims (*n* = 6), age below 18, and above 75 (*n* = 7), and medical history (*n* = 55). The rest of the patients were assessed for their eligibility based on laboratory test cutoff values-based. Further 205 patients were ineligible to participate, details of which are given in Fig. 1. Hence the final analysis is based on 244 participants randomized through permuted randomization plan (1:1) into Group A (*n* = 129) and Group B (*n* = 115) by statistician. The investigators enrolled the participants and assigned them to interventions. After screening and baseline visit, participants recruited in the groups were followed up at 6, 12, and 24 weeks’ time points. This study included the Holy month of Ramadan, and patients who fasted, were also asked to keep the data during Ramadan. The sample size was not achieved as physical follow-ups were suspended during the Covid-19 lockdown period and were conducted via teleclinics.

Patients were educated by diabetes educators, and diaries were provided to all patients at visit 1 (baseline) for recording and reporting of safety data on drug compliance, self-monitoring blood glucose, hypoglycemia, dehydration, hypotension, dietary habits, physical activities and hygiene practices, and other adverse events. The diaries were reviewed at visit 2 (6 weeks), visit 3 (12 weeks) and visit 4 (24 weeks). Patients were told to monitor their symptoms related to hypoglycemia like sweating, headache, trembling, etc. (described in the diary) if they found blood glucose level < 70 mg/dL. Similarly, they were briefed on monitoring symptoms of hypotension like dizziness, fainting, inability to concentrate, discomfort, SBP (less than 90 mmHg) and Diastolic blood pressure (DBP) (less than 60 mmHg) etc. Urinary tract infections were assessed by culture and sensitivity of the urine and Genital fungal infections were assessed after appropriate history and self-reported examination by the patient.

The physical activity index was also measured at visit 1 (baseline), which included evaluating the current exercise program by selecting the most appropriate score under each intensity, duration, and frequency category.

Diabetes Mellitus Quality of Life (DMQoL15) Satisfaction with Diabetes Control & adherence with self-care regimen questionnaire was used to collect data on Quality of life and satisfaction of all the participants [10]; the tool was implemented through interviewers at baseline visit and 3 follow up visits (6, 12 and 24 weeks respectively).

The study followed per-protocol analysis. Data were analyzed using SPSS version 15.0. Clinical characteristics, comorbid conditions, and laboratory results were compared between Group A and B at baseline. The normality of continuous variables was assessed using Shapiro Wilk tests. Mean with standard deviation and median with interquartile range is reported according to the distribution. The student’s t-test or Mann Whitney U test assessed the significant difference between two specific visits. We analyzed the changes of dependent variables from baseline to 6 months in HbA1c, FBG, weight, BMI, SBP, Alanine aminotransferase (ALT), high-density lipoprotein (HDL), and low-density lipoprotein (LDL) using repeated measure ANOVA. Frequency and percentages were reported for categorical variables using Chi-square or Fisher’s exact test depending upon cell count assumption. A *p*-value of < 0.05 was considered as a cut-off for a significant difference between the two groups.

The study was approved by the institutional review boards from Postgraduate Medical Institute Hayatabad Medical Complex Peshawar (ERC No. 5579/Dy.Reg./PGMI) and the National Institute of Cardiovascular Diseases (ERC-56/2019), Karachi. Informed consent was obtained from all participants at the time of enrolment after a thorough explanation of the study. This study was performed in accordance with all relevant and applicable guidelines and regulations.

## Results

Out of 244 participants recruited in the trial, 129 (52.8%) were randomized to the Empagliflozin arm (Group A) and 115 (47.2%) in the standard care arm (Group B) (Fig. 1).

Baseline characteristics of age, gender, BMI, duration of type 2 diabetes, and smoking status were similar between the two arms (Table 1). There were more females in Group A as compared to Group B (53.5% vs. 43.5%). Participants in Group A were overweight (88.4% vs. 82.6%) and had a slightly higher median duration of diabetes history. Comorbid conditions, i.e., hypertension, obesity, concomitant medication history, and biochemical profile of participants were statistically insignificant between the two arms.

**Table 1:** Baseline characteristics of participants recruited in the trial (*n* = 244)

Variables	Group A (***n*** = 129)	Group B (***n*** = 115)	***p***-value
**Gender**			
Male	60 (46.5%)	65 (56.5%)	0.118
Female	69 (53.5%)	50 (43.5%)	
**Age –years**	50.1 ± 10.2	50 ± 10.6	0.971
**BMI**			
BMI –kg/m^2^	29.6 ± 4.9	28.9 ± 4.9	0.331
Normal (18.5 to < 25.0)	5 (3.9%)	11 (9.6%)	0.198
Overweight (25.0 to < 30.0)	10 (7.8%)	9 (7.8%)	
Obese (≥ 30.0)	114 (88.4%)	95 (82.6%)	
**Duration of Type 2 DM-years**	4.1 ± 4.3 (2; 1–22)	3.7 ± 4.7 (1; 0.3–33)	0.572
**Smoking Status**			
Never	112 (86.8%)	98(85.2%)	0.937
Ex-Smoker	10 (7.8%)	10(8.7%)	
Smoker	7 (5.4%)	7(6.1%)	
**Comorbid Conditions**			
Hypertension	38 (29.5%)	29 (25.2%)	0.459
Dyslipidemia	25 (19.4%)	17 (14.8%)	0.342
Obesity	23 (17.8%)	16 (13.9%)	0.405
Non-alcoholic fatty liver disease	4 (3.1%)	8 (7%)	0.164
Cardiovascular Disease	3 (2.3%)	1 (0.9%)	0.371
Retinopathy	2 (1.6%)	2 (1.7%)	0.908
Neuropathy	12 (9.3%)	12 (10.4%)	0.767
Nephropathy	1 (0.8%)	–	0.344
**Concomitant Medication History of Study Participants**			
**Glucose Lowering Agents**			
Biguanides	111 (86%)	85 (73.9%)	0.027
Sulphonyl urea	32 (24.8%)	37 (32.2%)	0.202
Dipeptidyl-peptidase 4 (DPP4) inhibitor	59 (45.7%)	59 (51.3)	0.385
Insulin	12 (9.3%)	12 (10.4%)	0.767
**Vitals of Study Participants**			
Heart Rate-bpm	82.5 ± 9.3	83.2 ± 10.2	0.528
Systolic Blood Pressure-mmHg	128.2 ± 16.2	128.3 ± 15.2	0.955
Diastolic Blood Pressure-mmHg	81.4 ± 9.7	81.4 ± 8.7	0.947
**Baseline Biochemical Profile of Study Participants**			
Alanine Aminotransferase -IU/L	41.9 ± 27.0 (34; 11–154)	43.4 ± 29.6 (32; 7–161)	0.404
Aspartate Aminotransferase -IU/L	31.4 ± 14.1 (28; 12–93)	32.3 ± 15.8 (27; 13–111)	0.398
Alkaline phosphatase -IU/L	89.7 ± 25.0	88.7 ± 26.8	0.545
eGFR (mL /min /1.73m^2^)	100.9 ± 26.3	101.0 ± 25.5	0.956
Creatinine -mg/dl	0.8 ± 0.213	0.8 ± 0.2	0.929
Urea Nitrogen -mg/dl	14.3 ± 5.3 (13; 4–33)	14.2 ± 5.4 (13; 5–37)	0.809
Glycated Hemoglobin (HbA1c)			
NGSP	8.3% ± 0.9	8.3% ± 0.9	0.659
IFCC	67 mmol/mol	67 mmol/mol	
Fasting glucose (venous)-mg/dl	149.8 ± 45.3 (143; 70–355)	148.4 ± 43.0 (139; 58–344)	0.796
Total cholesterol-mg/dl	169.2 ± 40.6	174.5 ± 43	0.324
Low-density lipoprotein cholesterol-mg/dl	110.3 ± 40.8	114.9 ± 42.5	0.394
High-density lipoprotein cholesterol-mg/dl	37.7 ± 9.5 (36; 18–80)	37.1 ± 8.6(36; 18–60)	0.637
Triglycerides-mg/dl	181.0 ± 120.2 (154; 53–960)	196.2 ± 118.9 (181; 50–1058)	0.321
Hemoglobin-g/dl	13.5 ± 1.8	13.7 ± 2.3	0.323
White Blood Cells-10^3^/μL	8.7 ± 2.2	8.1 ± 2.0	0.026

Group A-Empagliflozin; Group B-Standard Care

*eGFR* Estimated glomerular filtration rate

During the study period, a total of 24 participants reported adverse events, 8 (7.4%) in Group A and 16 (15.8%) in Group B as part of drug safety analysis (Table 2). There were 4 (3.7%) participants who reported adverse events more than once during the study period in Group A, whereas 9 (8.9%) reported in Group B. Two patients in each group were discontinued due to the adverse events. Table 2 presented the number of events reported by participants wherein hypoglycemic events were considerably high in the standard care group. In contrast, positive urine culture, fungal infection, dehydration and hypotension, were comparable between two groups.

**Table 2:** Comparison of adverse events between Empagliflozin and standard care groups during the study period

Adverse Events	Group A	Group B	*P*-value
	(***n*** = 108)	(***n*** = 101)	
**Hypoglycemic events**			
Yes	6(5.6)	10(9.9)	0.238
No	102(94.4)	91(90.1)	
**Dehydration**			
Yes	3 (2.78)	3 (2.97)	0.934
No	105(97.2)	98(97.0)	
**Hypotension**			
Yes	1 (0.93)	1 (0.99)	0.962
No	107(99.1)	100(99.0)	
**UTI**			
Positive	6 (5.56)	7 (6.93)	0.681
Negative	102(94.4)	94(93.1)	
**Fungal Infection**			
Yes	–	2 (1.98)	0.142
No	108(100)	99(98.0)	
Treatment Discontinuation due to AE*	2 (1.6)	2 (1.7)	0.908

Group A-Empagliflozin; Group B-Standard Care

Values are presented in n (%). Chi-squared test was applied to determine the *P*-value considered significance at < 0.05 *AE Adverse events

**Fig. 2:**
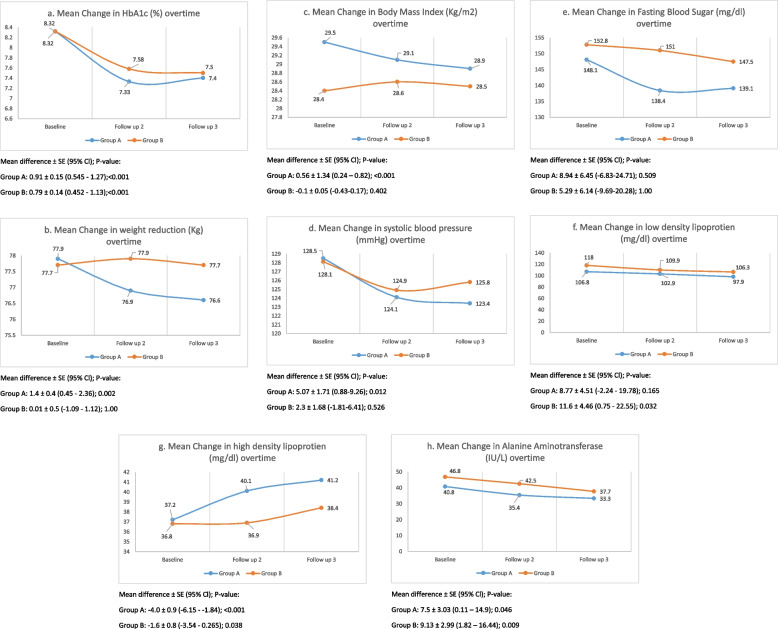
Effect of treatment overtime in among treatment groups (Empagliflozin and standard care) [HBA1c (**a**), weight (**b**), BMI (**c**) systolic blood pressure (**d**), FBS (**e**), LDL (**f**), HDL (**g**), ALT (**h**), overtime in treatment groups (Empagliflozin & standard care group). Two-factor ANOVA with repeated measures analysis was performed to determine the primary safety outcomes in all patients who completed the study. *P*-value < 0.05 was considered as the level of significance

**Fig. 3:**
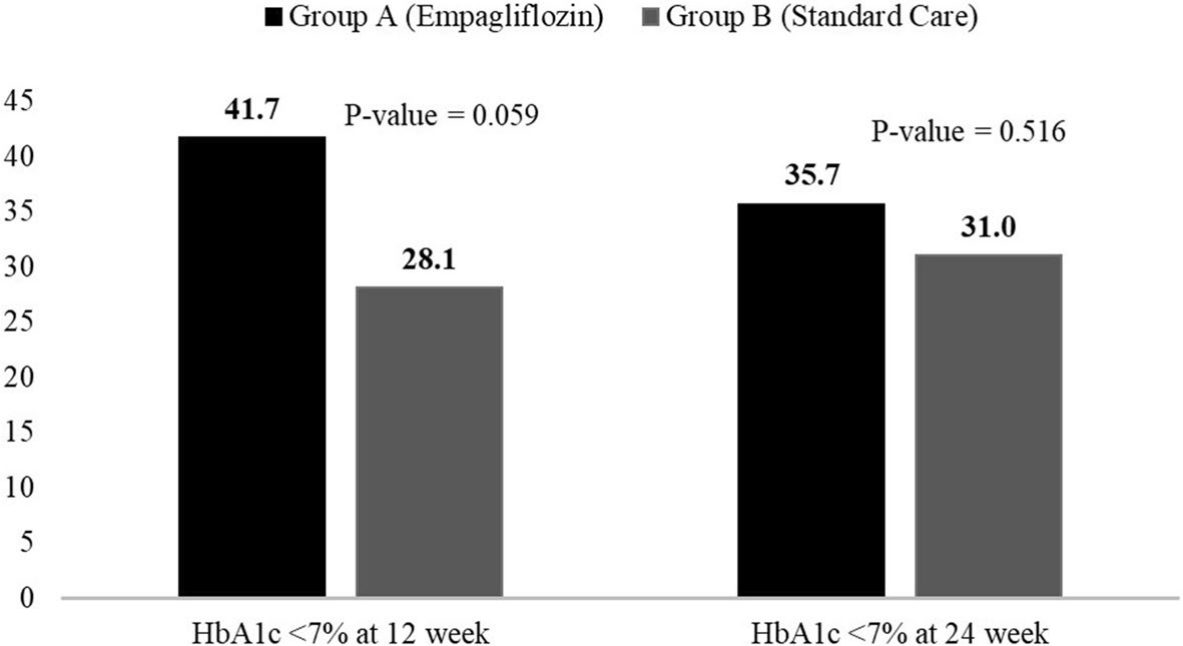
Percentage of patients achieved HbA1c < 7% /53 mmol/mol over time. For drug efficacy compared at 24 weeks’ time-point, reduction in mean HbA1c was significant in both groups, but the overall effect in reducing glycemic levels from baseline was significantly higher in Group A as compared to Group B (total effect difference: 0.91 ± 0.15 vs. 0.79 ± 0.14) (Fig. 3). A higher proportion of participants reached HbA1c < 7% (53 mmol/mol) at the 2nd and 3rd follow-up time point in Group A compared to Group B (41.7 and 28.1% vs. 35.7 31%, respectively. However, the HbA1c reduction is similar in both groups

Over the course of the trial duration, participants in Group A achieved a significant reduction in weight, (*P*-value = 0.002) BMI (*P*-value = 0.001), systolic blood pressure (*P*-value = 0.025), ALT levels (*P*-value = 0.046), HDL (*P*-value = < 0.001) but LDL (*P*-value = 0.165) was statistically insignificant as compared to Group B (Figs. 2 and 3).

Similarly, in patients with FBG > 100 mg/dl at baseline achieved FBG 100–120 mg/dl at follow up visit 2 and 3 were higher in Group A as compare to Group B 22.5% vs. 20% (*P*-value = 0.047) and 27.5% vs 19% (*P*-value = 0.534) respectively.

**Fig. 4:**
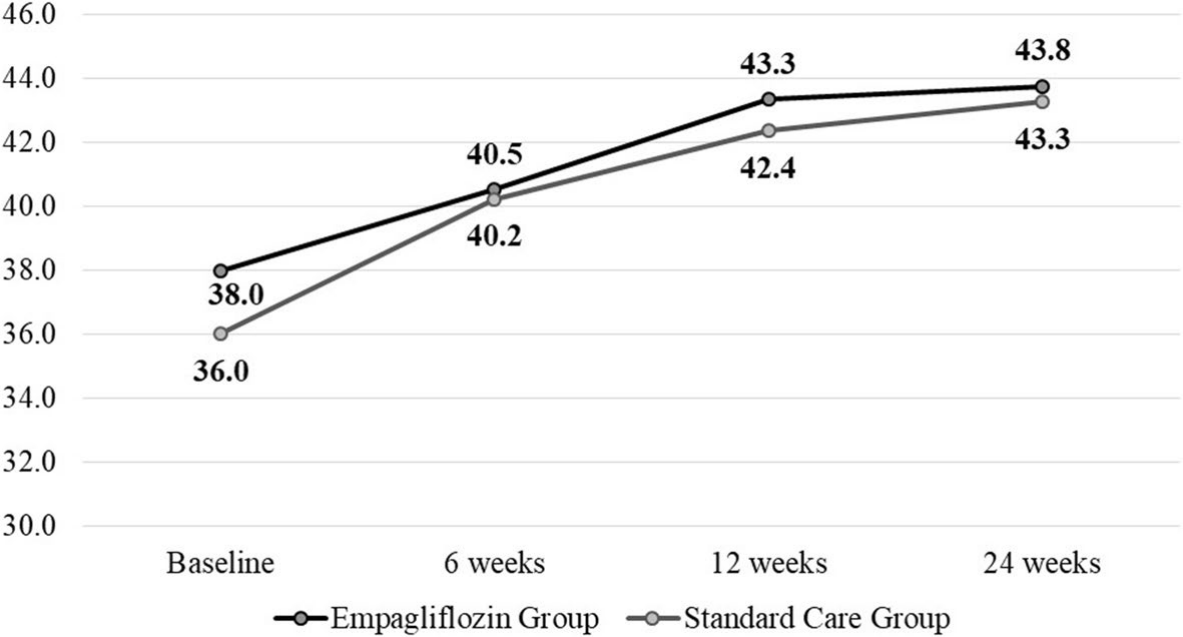
DMQOL15 Quality of life scores from baseline till 24 weeks follow up

There were only 5 patients who fasted during Ramadan, so analysis of those was not possible. There was a slight increase in Urea Nitrogen which was clinically significant (*P*-value = 0.005). The rest of the biochemical profile is shown in Table 3. Patients in Group A had slightly higher scores on DMQoL15 (Fig. 4).

**Table 3:** Difference in biochemical profile from baseline to 24 weeks

Laboratory Parameters	Group A (***n*** = 84)		Group B (***n*** = 87)	
	Mean Difference ± SE (95% CI)	***p***-value	Mean Difference ± SE (95% CI)	***p***-value
**ALP (IU/L)**	3.82 ± 2.46(−2.21–9.85)	0.376	3.62 ± 2.25(− 1.89–9.12)	0.338
**eGFR (mL/min/1.73m**^**2**^**)**	−1.58 ± 2.79(− 8.40–5.24)	1.000	−3.24 ± 3.14(10.92–4.43)	0.916
**Creatinine (mg/dl)**	0.03 ± 0.01(− 0.01–0.06)	0.145	0.02 ± 0.02(0.04–0.07)	1.000
**BUN (mg/dl)**	1.60 ± 0.51(0.40–2.87)	0.005	1.54 ± 0.58(0.12–2.95)	0.028
**TC (mg/dl)**	5.78 ± 4.26(−4.60–16.2)	0.534	5.21 ± 4.41(−5.52–15.97)	0.721
**TG (mg/dl)**	17.83 ± 13.30(−14.70–50.39)	0.553	−1.94 ± 15.20(− 38.98–35.10)	1.000
**Hb (g/dl)**	1.51 ± 1.47(−5.10–2.10)	0.930	−1.25 ± 1.62(− 5.20–2.70)	1.000
**WBC (10**^**3**^**/**μL**)**	0.42 ± 0.23(−0.14–0.98)	0.227	0.50 ± 0.19(0.04–0.96)	0.029
**RBC (10**^**6**^**/μL)**	0.08 ± 0.07(−0.24–0.08)	0.664	0.10 ± 0.09(− 0.12–0.31)	0.798
**Hematocrit (%)**	−0.80 ± 0.57(− 2.19–0.60)	0.501	0.39 ± 0.71(− 1.34–2.11)	1.000
**MCV (fL)**	0.55 ± 1.17(− 2.33–3.42)	1.000	− 0.31 ± 1.12(− 3.03–2.42)	1.000
**Platelets (10**^**3**^**/**μL**)**	31.1 ± 7.78(12.05–50.05)	< 0.001	25.33 ± 6.34(−9.84–40.82)	< 0.001

*BUN* Blood Urea Nitrogen, *eGFR* estimated glomerular filtration rate, *ALP* Alkaline phosphatase, *TC* Total cholesterol, *TG* Triglycerides, *Hb* Hemoglobin, *MCV* Mean corpuscular volume, *WBC* White Blood Cells, *RBC* Red Blood Cells Group A-Empagliflozin; Group B-Standard Care

Values are presented in n (%) or mean ± Standard deviation (median; range). Two-factor ANOVA with repeated measures analysis was performed to determine the mean difference in laboratory parameters in all patients who completed the study. *P*-value < 0.05 was considered as the level of significance

## Discussion

This study addresses the safety and efficacy of Empagliflozin in a Pakistani Muslim population with T2DM. To the best of our knowledge, this is the first study conducted in the Pakistani Muslim population focusing on the regional safety and efficacy knowledge gap of Empagliflozin use in the type 2 diabetes population. Considering the results of the follow-up data, we found Empagliflozin better in terms of drug safety, comparable Quality of life, and satisfaction with type 2 diabetes control. However, the HbA1c reduction is similar in both groups.

Assessing the drug safety, Empagliflozin fared well in comparison to standard care groups in terms of lesser adverse events. Compared with other regional and global estimates, this is different from what was reported in the meta-analysis of 25 randomized controlled trials assessing the safety and efficacy of Empagliflozin [11]. In our study, Empagliflozin showed lower cases of urinary tract infections in the participants less occurrence of hypotension and hypoglycemia than standard care groups. This could be attributed to different (wet hygiene) practices amongst the Muslim population of Pakistan. Empagliflozin used at higher doses of 50 mg has previously shown an increased chance of developing urinary tract infections [12]. However, in this same study, there was also an increased chance of using the drug at lower doses. Another study of a total of 7028 patients underwent randomization over a period of 3 years from 2010 to 2013, examining the effects of Empagliflozin compared to placebo on cardiovascular morbidity and mortality in patients with type 2 diabetes at high risk of cardiovascular events who were receiving standard care. Regarding the proportion of patients who had adverse events, they were similar in both Empagliflozin and Placebo groups. Urosepsis was reported in 0.4% of patients in the Empagliflozin group and 0.1% in the placebo group. There was no increase in the overall rates of urinary tract infections [13, 14].

Yet another study including 899 patients investigated the long-term efficacy and safety of Empagliflozin monotherapy compared with placebo and sitagliptin (a dipeptidyl peptidase-4 inhibitor) in the drug naïve patients with T2DM. Events consistent with urinary tract infections were reported in a similar proportion of patients in each treatment group. Urinary tract infections were mild or moderate in intensity except in one patient on Empagliflozin 25 mg and one on sitagliptin. In line with previous studies of Empagliflozin [15], there was no higher risk of urinary tract infections in patients with Empagliflozin in this study. This is in line with our study, although a different population.

Despite having a higher proportion of the overweight and obese population, Empagliflozin performed better and produced favorable results for controlling glycemia, reducing weight, and overall cardio-physiological profile compared to the standard care group. The findings of our study are comparable to the other regional populations [16].

Empagliflozin has previously been reported to significantly reduce 24-hour ambulatory systolic BP versus placebo by weeks 12 and 24. With a reduction in diastolic blood pressure in black patients with type 2 diabetes mellitus, Empagliflozin reduced glycohemoglobin, body weight, and blood pressure. The effect of Empagliflozin on blood pressure was favorable from 12 to 24 weeks, suggesting that Empagliflozin may be beneficial for this high-risk population [17]. SGLT2 inhibitors are known to cause natriuresis associated with glycosuria and volume depletion, which can cause a slight increase in blood urea nitrogen (BUN). This effect was seen in the Empagliflozin group in our study. This effect is usually transient based on previous studies [18].

Another landmark study has shown a definitive protective effect of Empagliflozin with reduced hospitalization and death due to cardiac events [13]. Comparing these, our study carried out in the Pakistani Muslim population has shown promising values with Empagliflozin in type 2 diabetes mellitus patients with a similar reduction in systolic blood pressure, improved physiological indicators, and weight loss as previously reported in a similar population, all of which play a significant role in reducing possible adverse cardiac events. Significant improvements in HbA1c, glycemic levels, and overall weight indicate the sustained glycemic effects of Empagliflozin, which significantly reduced HbA1c and fasting plasma glucose, a phenomenon previously reported in the local population [19]. There was also a considerably higher percentage of patients that achieved the glycemic target of HbA1c < 7% (53 mmol/mol) compared to placebo, similar to what was observed in previous studies [20]. Participants receiving Empagliflozin also scored higher on the quality-of-life scale from baseline till 24 weeks follow up.

The safety and efficacy of Empagliflozin demonstrated by our study with lower Urinary tract infection (UTI) events might be due to wet hygiene practices among Pakistani Muslim population. Furthermore, higher patient satisfaction, and Quality of life in patients compared to the standard care are the strengths of this nationwide multicenter randomized controlled trial covering wide variety of subjects in terms of demographics and climatic variations. There was also a high adherence to Empagliflozin regimen among patients from baseline to 24 weeks, despite ongoing pandemic and challenges in patient follow-up during national COVID restrictive protocols (more than 80% follow up at 6-week visit) leading to early closure of enrollments.

## Limitations

The first limitation of our study is the use of Empagliflozin if given in variable doses such as 10 and 25 mg to compare the dose-response relationship for safety, efficacy, and factors related to patient experience. Secondly, as the study follow-up period included the Holy month of Ramadan, due to a small number of patients who fasted, analysis was not conclusive. Likewise, important data on the possible risk of Diabetic ketoacidosis could not be ascertained in safety. Thirdly, education on wet and dry hygiene was provided to the participants in this trial (as part of the clinical regimen), which might have reduced the frequency of fungal infections and UTIs, but this factor was not gauged in the present study. Finally, due to the short course of the study, we could not record microalbuminuria data which was another limitation.

## Conclusion

Empagliflozin was found to be a safe drug as compared to standard of care in Pakistani Muslim T2DM individuals. Empagliflozin is as effective as standard care but achieves glycemic control with weight loss and significant blood pressure-lowering effect, especially systolic blood pressure, compared to standard care.

## Data Availability

The datasets generated and/or analyzed during the current study are available in the Mendeley Data repository, https://data.mendeley.com/datasets/9g3bvkyjzw/1.

## Abbreviations

BMI: Body mass index
HbA1c: Glycosylated hemoglobin
SBP: Systolic blood pressure
SLT2: Sodium-glucose Cotransporter-2
T2DM: Type 2 Diabetes mellitus
QoL: Quality of life
DBP: Diastolic blood pressure
DMQoL15: Diabetes Mellitus Quality of Life
ALT: Alanine aminotransferase
HDL: High-density lipoprotein
LDL: Low-density lipoprotein
UTI: Urinary tract infection
